# Pleiotropic Effects of IL-2 on Cancer: Its Role in Cervical Cancer

**DOI:** 10.1155/2016/2849523

**Published:** 2016-05-12

**Authors:** Arturo Valle-Mendiola, Adriana Gutiérrez-Hoya, María del Carmen Lagunas-Cruz, Benny Weiss-Steider, Isabel Soto-Cruz

**Affiliations:** ^1^Molecular Oncology Laboratory, Cell Differentiation and Cancer Research Unit, FES Zaragoza, National University of Mexico, Batalla 5 de Mayo s/n, Colonia Ejército de Oriente, 09230 Mexico City, DF, Mexico; ^2^Cátedra CONACYT, FES Zaragoza, National University of Mexico, Mexico City, DF, Mexico

## Abstract

IL-2 receptor (IL-2R) signalling is critical for normal lymphocyte proliferation, but its role in cervical cancer is not fully understood. The receptor is composed of three chains: IL-2*α*, IL-2*β*, and IL-2*γ*. Intracellular signalling is initiated by ligand-induced heterodimerization of the IL-2*β* and IL-2*γ* chains, resulting in the activation of multiple intracellular kinases. Recently, IL-2R was shown to be expressed on nonhaematopoietic cells, especially on several types of tumour cells. However, the function of this receptor on malignant cells has not been clearly defined. The expression of IL-2R and the production of IL-2 in cervical cancer cells have been documented as well as expression of molecules of the JAK-STAT pathway. In the current review we have highlighted the differences in the responses of molecules downstream from the IL-2R in normal lymphocytes and tumour cells that could explain the presence of tumour cells in an environment in which cytotoxic lymphocytes also exist and compete and also the effect of different concentrations of IL-2 that could activate effector cells of the immune system cells, which favour the elimination of tumour cells, or concentrations that may promote a regulatory microenvironment in which tumour cells can easily grow.

## 1. IL-2 and Its Receptor in Normal Cells

Interleukin 2 (IL-2) is a 15.5 kDa cytokine that is primarily produced by CD4^+^ T cells following antigen stimulation [1] and to a lesser extent by CD8^+^ cells [2], NK T cells [3], mast cells [4], monocytes [5], and myeloid dendritic cells (mDCs) [5, 6]. IL-2 is a key regulator of normal immune functions and is critical for the activation and subsequent amplification of the immune response following antigenic stimulation. Moreover, IL-2 promotes regulatory T cell development and constrains Th17 cell polarization [7–9]. To elicit these biological effects, IL-2 sends signals through the IL-2 receptor (IL-2R) complex. This complex is comprised of two essential signalling subunits (IL-2R*β* and IL-2R*γ*) and one affinity modulating subunit (IL-2R*α*). IL-2 can exert its effect on cells expressing either the intermediate-affinity (*K*_*d*_ = 10^−9 ^M) receptor dimer of IL-2R*β* and the common IL-2R*γ* chain or the high-affinity (*K*_*d*_ = 10^−11 ^M) trimeric IL-2R comprised of IL-2R*α*, IL-2R*β*, and IL-2R*γ* [7]. IL-2-induced heterodimerization of IL-2R*β* and IL-2R*γ* results in activation of the receptor-associated Janus tyrosine kinase (JAK) 1 and JAK3 through trans- or autophosphorylation [10, 11]. Subsequent tyrosine phosphorylation of the IL-2R*β* chain provides docking sites for effector molecules, including signal transducer and activator of transcription (STAT) 5a and STAT5b, via their Src homology 2 domains [12].

IL-2R*β* propagates signals following receptor-ligand engagement, thereby controlling the recruitment and activation of effector proteins, and is known to be phosphorylated on its tyrosine; this modification of the *β* chain has been studied extensively. However, the identification and putative regulatory roles for serine and threonine phosphorylation sites have not been fully characterized. Ruiz-Medina et al. [13] demonstrated that the phosphorylation of IL-2R*β* Thr450 was rapid (2.5 min), transient (peak at 15 min), and protracted compared with receptor tyrosine phosphorylation and occurred in multiple cell types, including primary human lymphocytes. Reconstitution assays demonstrated that Thr450 was important for the regulation of IL-2R complex formation, JAK3 recruitment, and the activation of Akt, ERK1/2, and transcriptionally active STAT5. These results provide the first evidence of the identification and functional characterization of threonine phosphorylation of an interleukin receptor.

Originally identified as the third subunit of the high-affinity IL-2 receptor, the common *γ*-chain (*γ*c) also acts as a nonredundant receptor subunit for a series of other cytokines that are collectively known as *γ*c family cytokines. Members of the *γ*c cytokine family (including IL-2) play distinct and nonredundant roles in the adaptive immune system, especially in the development and differentiation of T lymphocytes. The importance of *γ*c in the immune system is demonstrated by the profound phenotype associated with *γ*c gene deficiency that manifests as XSCID (X-linked severe combined immunodeficiency) [14]. Thus, *γ*c expression is a nonredundant requirement for lymphocytes and is especially critical for T cells in both humans and mice. Alternative splicing generates soluble *γ*c chains; in the case of IL-2R, all members produce soluble forms [15, 16]. A common feature shared amongst these soluble receptors is that they retain their affinity for their cognate cytokine ligands. Consequently, these secreted proteins can either compete with membrane cytokine receptors for ligand binding or sequester the cognate cytokine, thereby limiting its bioavailability [17].

## 2. Different Cell Types Express the IL-2R and Elicit a Response to IL-2

### 2.1. NK Cells

NK cells are specialized effector lymphocytes of the innate immune system that are capable of eliciting responses against pathogen-infected and tumour cells. Additionally, they are effectors of the rapidly acting innate immune system that mediates cellular cytotoxicity and produces cytokines and chemokines. NK cells are activated during the early phases of the immune response (a few hours after infection). The functions of NK cells are regulated by a balance between activating and inhibiting signals. These signals are transmitted by inhibitory receptors that bind class I major histocompatibility complex (MHC) molecules and activating receptors that bind ligands on tumours and pathogen-infected cells. In addition to surface receptors, cytokines such as IL-2, IL-12, IL-18, and type I IFNs have been shown to promote NK cell priming [18, 19].

Cytotoxic activity is one of the principal functions of natural killer cells and is critical for the innate immune responses against infected cancer cells. Recently, the IL-2R*α* subunit (CD25) was proposed as a candidate NK cell cytotoxicity marker [20].

The cross talk between dendritic cells (DCs) and NK cells has been described in the context of immune responses to infectious agents and tumours [21, 22]. Granucci et al. [23] showed that IL-2 produced early by bacterially activated mouse DCs played a fundamental role in the activation of NK cell-mediated immunity* in vitro* and* in vivo*. This result indicates that IL-2 is necessary for the regulation of innate immune responses in addition to its well-defined function in acquired immunity. Subsequent studies from the same workgroup showed that only TLR-dependent microbial stimuli associated with Th1 responses conferred DCs with the ability to activate NK cells, whereas stimuli associated with Th2 responses did not have this property [24].

IL-2 and IL-15 are two distinct cytokines with partially overlapping properties that are implicated in the development, homeostasis, and effector functions of NK cells. IL-15 mimics IL-2-induced T cell proliferation because it shares the *β* and *γ* subunits of the IL-2 receptor [25].

### 2.2. Regulatory T Cells

Regulatory T (Treg) cell-mediated suppression serves as a vital mechanism for the negative regulation of immune-mediated inflammation and features prominently in autoimmune and autoinflammatory disorders, allergies, acute and chronic infections, cancer, and metabolic inflammation [26]. IL-2 has been implicated in the generation and maintenance of Tregs, and these cells play an important role in the prevention of the development of systemic autoimmune diseases [27]. Treg cells appear to primarily constrain the expansion and development of conventional T cells into damaging effectors. Liu et al. observed pSTAT5-Treg clusters in the lymph node and proposed that TCR signalling was probably also required for the effective control of autoimmunity by promoting the colocalization of Treg cells with target T effectors on a dendritic cell platform; however, coclustering may only be optimized rather than solely mediated by a TCR-dependent mechanism. Indeed, autoreactive T cells are activated for cytokine production on a regular basis, with physically coclustering T cell receptor-stimulated Treg cells responding in a negative feedback manner to suppress incipient autoimmunity and maintain immune homeostasis [28]. IL-2R*α* (CD25) was one of the first useful markers identified for Tregs [29]. Therefore, IL-2 was hypothesized to be required for the development or function of Tregs [10, 30]. Notably, under physiological conditions IL-15 does not play a role in Treg development or contribute to the IL-2 signalling in Tregs that leads to downregulation of the IL-15R*α* chain, thereby rendering these cells much less responsive to IL-15 [31]. Malek and Lafaille were the first to show that IL-2 played an important role in both Treg development and function [32, 33].

### 2.3. B Cells

B cells play an important role in humoral immunity and are a pivotal component of the adaptive immune system because they are able to produce antibodies, present antigens, and secrete cytokines, such as IL-2. For example, B cell responses are guided by the integration of signals through the B cell receptor (BCR), CD40, and cytokine receptors. The main effect of IL-21 on B cell functions is focused on mature B cells in secondary lymphoid organs. IL-21 induces proliferation, class switching, or death in mature B cells depending upon the provision of accessory signals and antigenic stimuli [34, 35].

The common *γ* chain (*γ*c) binding cytokine IL-21 drives humoral immune responses via the STAT3-dependent induction of transcription factors required for plasma cell generation. Indeed, IL-21 strongly induced IL-2R*α* (CD25) in normal but not STAT3-deficient CD40L-stimulated naive B cells. Chromatin immunoprecipitation confirmed that IL-2R*α* was a direct target of STAT3. IL-21-induced CD25 expression was also impaired in B cells from patients with IL-2R*γ* or IL-21R mutations, confirming a requirement for intact IL-21R signalling in this process. IL-2 increased plasma blast generation and immunoglobulin secretion from normal but not CD25-deficient cells; these results demonstrate that IL-21 sensitizes B cells to the stimulatory effects of IL-2 via STAT3. Thus, IL-2 may play an adjunctive role in IL-21-induced B cell differentiation. A lack of this secondary effect of IL-21 may amplify humoral immunodeficiency in patients with mutations in STAT3, IL-2R*γ*, or IL-21R due to impaired responsiveness to IL-21 [36].

### 2.4. Dendritic Cells

Dendritic cells are specialized antigen-presenting cells and essential mediators of immunity and tolerance. This group of cells is heterogeneous in terms of cell surface markers, anatomic locations, and functions [37]. DCs are crucial for sensing pathogens and triggering immune responses. Upon activation by pathogen-associated molecular pattern (PAMP) ligands, GM-CSF myeloid DCs (GMDCs) secrete several cytokines (including IL-2) and express the IL-2R (*β* chain) at the steady state. Moreover, DCs employ the IL-2-mediated STAT5 signalling axis to promote the death of PAMP-stimulated GM-DCs to maintain immune tolerance and prevent autoimmunity [38]. In particular, Wuest et al. demonstrated that mDCs and antigen-experienced T cells secreted IL-2 into the mDC-T cell interface in an antigen-specific manner. Moreover, mDCs provide IL-2R*α* (CD25) to primed T cells in trans to facilitate early high-affinity IL-2 signalling [39]. DCs also induce IL-2 production by the *β*-glucan receptor Dectin-1 (a yeast binding C-type lectin that synergizes with TLR2) [40, 41] or zymosan [6, 42]. Zelante et al. demonstrated a crucial role for* Aspergillus*-induced IL-2 production by lung dendritic cells [43].

Different subtypes of DCs (including activated plasmacytoid dendritic cells (pDCs)) express IL-2R*α* (CD25). Naranjo-Gómez et al. demonstrated that both CpG and CD40L induced the upregulation of IL-2R*α* in pDCs within the first 6 h of activation. This acquisition of IL-2 responsiveness by pDCs may be important in the context of antigen presentation. When T cells recognize an antigen on DCs, the interaction is prolonged and IL-2 is produced. The ability of pDCs to respond to IL-2 may contribute to ongoing T cell activation by providing additional cytokines such as TNF or IL-4 [44].

More recently, DCs were shown to express the three subunits of the IL-2R, and activation with IL-2 induced STAT5 phosphorylation. This activation increases the ability of DCs to activate helpless CD8^+^ T cells. Furthermore, IL-2 induces the functional maturation and activation of monocyte-derived DCs through the IL-2 signalling pathway [45].

### 2.5. Monocytic Cells

Human monocytes play important roles in the immune response as immunomodulators, antigen-presenting cells, and effector cells. IL-2 and IFN-*γ* are powerful activators of tumoricidal human monocytes* in vitro*. These cells respond to IL-2 with hydrogen peroxide production, TNF-*α* induction, and microbicidal and tumoricidal activity [46].

Bosco et al. [47] demonstrated that resting monocytes constitutively expressed IL-2R*β*. This group provided the first evidence of the expression of IL-2R*γ* in monocytes and its modulation by IL-2 and IFN-*γ* through posttranscriptional mechanisms. In contrast, the inhibition of constitutive and IL-2-induced IL-2*γ* expression suggests that TGF-*β* should suppress the activation of monocytes by IL-2 if the gamma chain is required for their response to IL-2. TGF-*β* was shown to inhibit IL-6 induction by IL-2 in fresh monocytes and the IL-2-induced activation of monocytes to a cytotoxic stage; TGF-*β* also inhibited IL-2 binding to the surface of human monocytes [48]. Interestingly, the dose response of the downregulation of IL-2-induced *γ* chain expression by TGF-*β* correlated with the inhibition of IL-2-induced cytotoxicity. The downregulation of *γ* chain expression by TGF*β* is associated with the inhibition of the response to IL-2 and therefore may represent an important regulatory mechanism controlling the activation of monocytes by IL-2 [48, 49].

### 2.6. Nonimmune Cells

The expression of IL-2R is not limited to immune cells; in fact, human intestinal epithelial cells express functional receptors for IL-2 (excluding IL-2R*α*) [50]. The expression of IL-2R (and other members of the *γ*c family) on intestinal epithelial cells may give a high plasticity pathway to intestinal epithelial cell-lymphocyte interactions, which may prove critical for the function of the mucosal immune system [50, 51].

Gerritsma et al. [52] demonstrated that human proximal tubular epithelial cells (PTEC) expressed the high-affinity IL-2R, which might be involved in the regulation of the production of complement component C3, which is found during renal inflammation.

Moreover, a functional receptor (*β* and *γ* chains) is expressed in fibroblast-like synoviocytes (FLSs) and dermal fibroblasts (DFs), with FLSs expressing four times more receptors than DFs. This IL-2R expression has a particular function: in the inflamed synovium, activated T cells migrate and T cell-derived IL-2 may activate FLSs to secrete MCP-1, thereby recruiting macrophages into the synovium and helping to perpetuate inflammation [53].

Additionally, human fibroblasts express functional IL-2R (IL-2R*α*, IL-2R*β*, and IL-2R*γ*). These observations suggest that the range of cellular targets of IL-2 is broader than originally determined. Thus, IL-2 may integrate fibroblasts and monocytes into a coordinated response in the connective tissue that is initiated by T lymphocytes [54, 55].

## 3. IL-2 Signalling

The function and structure of the IL-2R have been well characterized in lymphocytes. IL-2R functions as a necessary signal for cell proliferation and homeostasis of lymphocytes. The intracellular signalling pathway is initiated by the ligand-induced heterodimerization of IL-2R*β* and IL-2R*γ*, which results in the activation of multiple kinases, including members of the JAK family, members of the Src family, and members of the Syk/ZAP70 family [8].

IL-2R is associated with the members of the Janus kinase family, including JAK1 and JAK3. These molecules are rapidly phosphorylated upon ligand binding and play critical roles in signalling downstream of the cytokine receptors [56, 57].

In normal cells, JAK activation occurs in a sequential manner: JAK3 activates JAK1 [8, 58, 59], and the presence of both molecules is necessary for the correct activation of the IL-2 signalling pathway. Additionally, JAK1 is the required partner of JAK3 for the induction of survival signals [60]. The primary JAK substrates are members of the signal transducer and activator of transcription (STAT) family; these substrates are phosphorylated by JAKs to convey the proliferation signal to the cell nucleus [61]. Once phosphorylated, the molecules dimerize and are able to translocate into the nucleus, bind DNA, and induce gene expression.

**Figure 1:**
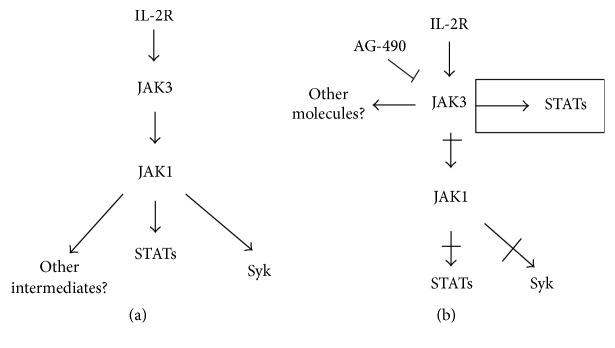
Model of the IL-2R signalling pathway. Zhou et al. [8] propose the model as follows: (a) the first molecule activated is JAK3 and the next molecule in the pathway is JAK1; Syk functions as a downstream element of the JAKs in IL-2 signalling. JAK3 may regulate Syk and STAT activity indirectly via JAK1. Our model proposed for cervical cancer cells is shown in (b). The crossed arrows represent the steps that are not performed in cervical cancer cells but appear in the normal cell model. The rectangle shows a step that is not found in the normal cell model.

Cells isolated from mice depleted of JAK1 failed to respond to lymphopoietic cytokines [57]; moreover, IL-2 stimulated JAK3 to a larger extent than JAK1 in human lymphocytes [62]. A hierarchy exists in the activation of molecules. According to the model proposed by Zhou et al. [8], Lck functions in parallel with JAK3, whereas Syk functions as a downstream element of the JAKs in IL-2 signalling. JAK3 may regulate Syk catalytic activity indirectly via JAK1. However, IL-2-mediated JAK3/STAT activation is not dependent on Lck or Syk (Figure 1(a)). Additionally, JAK3 activation precedes JAK1 activation [58].

## 4. Cervical Cancer and IL-2

Cervical cancer is the second leading cause of cancer death in women worldwide, resulting in approximately 270,000 deaths each year [63]. Most cervical carcinomas are related to human papilloma virus (HPV) and have a stepwise progression starting from premalignant lesions; however, HPV is not sufficient for cervical carcinogenesis and tumour progression. Depressed immune responses have been frequently observed in cancer patients. The expression of the IL-2R and the production of IL-2 by tumour cells have been shown in a variety of human malignancies [9–12]. However, the role of IL-2 in cervical cancer is not fully understood. IL-2 has been widely used as a treatment for several tumours based on the finding that this cytokine promotes the proliferation and activity of cytotoxic lymphocytes. However, high doses of IL-2 must be used to obtain the desired therapeutic effect. According to our observations [64], high doses of IL-2 inhibit the proliferation of IL-2R-expressing tumour cells regardless of the cytotoxic function of the activated lymphocytes. This response might be responsible for the observed tumour reduction. Unfortunately, the high doses needed for treatment are very toxic to humans and limit the application of this treatment. To overcome this high toxicity, several protocols have been developed using low doses of IL-2, but as previously shown caution should be exercised when tumours express IL-2R because the cells may be induced to proliferate. Thus, a useful preventive measure before considering treatment with low doses of IL-2 is to test for the presence of IL-2R.

## 5. IL-2 Expression and Signalling in Cervical Cancer Cells

As mentioned above, the expression of IL-2R is not limited to haematopoietic cells. This receptor has been found to be expressed on nonhaematopoietic cells, especially on several types of tumour cells such as melanoma, human squamous cell carcinomas, and cervix, breast, and lung cancers [9–12, 65–67].

Normal cervical cells do not express IL-2R, but the expression of IL-2R in cervical cancer cells has been well documented by our own group [11, 12, 65]. Some reports indicate that this phenomenon is related to the lesion progression. For example, Mindiola et al. demonstrated that the expression of IL-2, IL-2R, and IL-10 in cervical tissue may play a role in the development of cervical intraepithelial dysplasia [68], whereas the expression of IL-2 is associated with cell proliferation in cases of squamous cell carcinomas of the head and neck (SCCHN) [69].

In cervical cancer, the aberrant expression of molecules is not restricted to IL-2 or IL-2R. This type of cancer has been reported to express a variety of molecules, some of which are associated with the progression of HPV-associated cervical cancer, such as STAT5A [70], hypoxia-inducible factor 1*α* (HIF-1*α*), and glucose transport protein 1 (GLUT1) [71]. Other molecules reported to be present in cervical cancer cells include c-Kit, HER2 [72, 73], JAK3, STAT5 [12, 64], JAK1 [64], MICA, MICB, NKG2D [74], HER3 (Zerecero-Carreon, unpublished data), STAT3, Syk, and Lck (unpublished data).

This normal signalling pathway is altered in transformed cells. For example, STAT5 is constitutively activated in several solid tumours [12, 75–77]. This activation is consistent with the reported oncogenic functions of STAT5 in a variety of haematopoietic malignancies and supports a role for its activation in solid tumours [75, 76, 78, 79]. Nonetheless, in some diseases STAT3 or STAT5 expression indicates a favourable prognosis, such as breast and nasopharyngeal cancer [77, 80]. In contrast, constitutively active mutants of STAT5 have been shown to be oncogenic* in vivo* and* in vitro* [81], and STAT3 serves as an oncogene [82]. These findings suggest that STAT activation is important for the maintenance of the malignant phenotype of cancer cells.

The JAK1 and JAK3 responses in cervical cancer cells are different from the responses reported for normal lymphocytes. In this model, the two molecules (JAK1 and JAK3) are present but only JAK3 is constitutively phosphorylated; moreover, JAK1 is present but is not phosphorylated in response to IL-2 [64]. In contrast, in normal lymphocytes the presence and activation of both JAKs are necessary for the correct activation of the IL-2 pathway, and JAK1 is the partner of JAK3 for the activation of survival signals [83]. In normal lymphocytes, IL-2 primarily stimulates JAK3. The same phenomenon occurs in the YT cell line [59], where JAK3 is the first molecule activated [8]; moreover, thymocytes from null mice that lack JAK1 fail to respond to IL-2 [84].

The characteristics of constitutive JAK3 phosphorylation and the lack of JAK1 phosphorylation in cervical cancer cells have been previously documented in transformed and malignant lymphocytes. JAK1 activation is not required for JAK3 and STAT5 activation in MOLT4 cells [84], and JAK3 overexpression results in its own activation. Moreover, JAK3 but not JAK1 is constitutively phosphorylated in mycosis fungoides and its leukaemic variant Sezary syndrome [85, 86].

These results suggest that the lack of JAK1 phosphorylation and the constitutive activation of JAK3 might be characteristics of an IL-2R-expressing transformed cell; therefore, it is possible to speculate that the JAK3 kinase may be “more important” in IL-2 signalling than JAK1 [64] (Figure 1(b)). JAK3 alone may be capable of initiating the signals induced by IL-2 even if JAK1 is not activated in cervical cancer cells. Because JAK3 is the only active kinase, it is likely to be the main molecule responsible for initiating the response to IL-2 without the involvement of JAK1 as usually occurs in lymphocytes [64].

## 6. Roles of IL-2 in the Immunoregulatory and Antitumour Effects on Cervical Cancer

**Figure 2:**
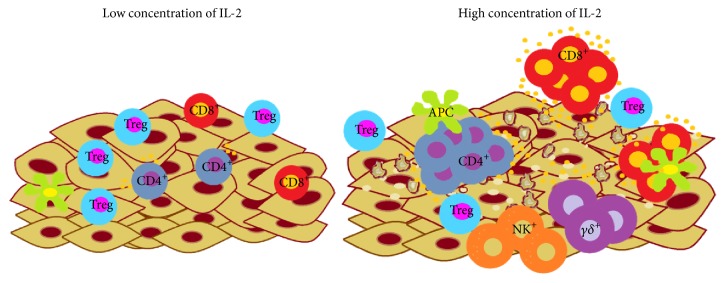
Concentration of IL-2 and activation of tumour-infiltrating lymphocytes. Low concentrations of IL-2 may promote the regulatory microenvironment and tumour growth. High concentrations of IL-2 activate immune system cells, such as CD8^+^, CD4^+^, and *γδ* T cells and NK cells; these cells produce proinflammatory cytokines, such as IFN-*γ*, and eliminate tumour cells.

Cytokines can have both negative and positive effects on cells undergoing carcinogenesis. The promotion and progression phases of carcinogenesis may be affected by autocrine loops involving cytokines with growth factor activities, such as IL-2. Accordingly, the hypothesis of our group is that high concentrations of IL-2 activate immune system cells, such as CD8, CD4 and *γδ* T cells, and NK cells, to favour the elimination of tumour cells. Conversely, low concentrations may promote a regulatory microenvironment that allows tumour cells to easily grow (Figure 2). This hypothesis is supported by our data. We previously described differences in the responses of molecules downstream of IL-2R in normal lymphocytes and tumour cells. The lack of JAK1 phosphorylation, the activation of JAK3 and STAT5 in response to low doses of IL-2, and the opposite effect in response to high doses of IL-2 indicate different mechanisms for the IL-2R transduction pathway that may explain the presence of tumour cells in an environment where cytotoxic lymphocytes also exist and compete for IL-2 [64].

**Figure 3:**
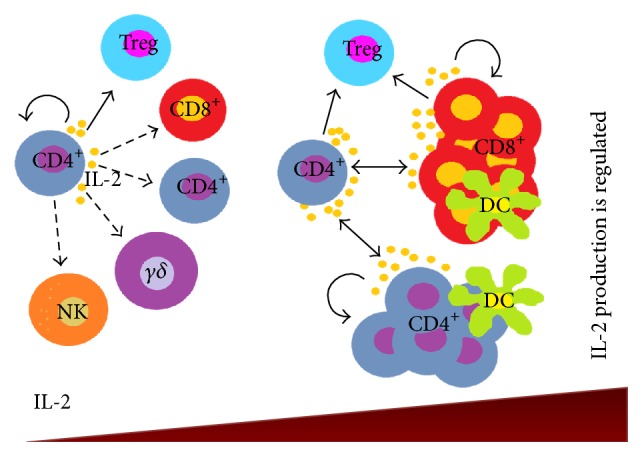
IL-2 concentration and homeostasis. Under steady state conditions, interleukin 2 is primarily produced by activated T cells, particularly CD4^+^ T cells. Then, the secreted IL-2 is consumed by Treg cells and adjacent activated CD4^+^, CD8^+^, and *γδ* T cells and NK cells (cells with functional high-affinity IL-2 receptors). Binding of IL-2 to its receptor initiates activating and/or mitogenic signals and induces the differentiation of naïve T cells into effector and memory T cells. The survival of Ag-selected T cells is essential for the maintenance of Treg cells. Additionally, DCs are able to produce IL-2 in response to the recognition of some pathogen-associated molecular patterns, which favours the proliferation of specific T cells. IL-2 production is regulated by silencing the IL-2 gene to prevent the apoptosis of these cells.

IL-2 is essential for naïve T cell differentiation into effector and memory T cells [87], the survival of antigen-selected T cells, and the maintenance and development of Treg cells (Figure 3). The absence of IL-2 or IL-2R in mice causes the development of a lethal autoimmunity that lacks a negative regulatory function due to the absence of Treg cells. In humans, defects in the IL-2 or IL-2R pathway are associated with autoimmune diseases such as type 1 diabetes or systemic lupus erythematosus. Nevertheless, these patients have a potential decrease in cytotoxic T lymphocyte activity and are more susceptible to intracellular infection because IL-2 is necessary for the development of CD8^+^ T cell and NK cell cytotoxic activity [88, 89]. Conversely, IL-2 production is regulated by silencing some IL-2 gene transcription factors, and persistent stimulation of T cells can induce the expression of the death receptor FAS (CD95), which can promote the apoptosis of these cells. We need to better understand the physiological role of IL-2 in immunity and self-tolerance because IL-2 signalling can promote immunity or inhibit and promote tolerance [90]. We propose that the modulation of IL-2 can be used to selectively activate T cell subsets towards clinical use, although the use of IL-2 in the clinic remains controversial. Evidence that supports the use of IL-2 administration in cancer treatment has been reported. For example, BubenÌk et al. showed results that indicated the therapeutic effects of this cytokine and suggested that local IL-2 administration was beneficial for cancer immunotherapy [91, 92]. However, the optimal form of administration and concentration are unclear because IL-2 is a pleiotropic cytokine and its effects depend upon the concentration, cell type, and microenvironment.

Several studies have shown that IL-2 signals are active in different cell subpopulations. Somewhat paradoxically, low doses of recombinant IL-2 have been used for Treg cell-based immunosuppressive strategies against immune pathologies, whereas high doses of IL-2 have shown some success in stimulating antitumour immune responses [90–92]. This finding indicated that the concentration of IL-2 was very important for the development of the immune response. The involvement of the host immune system in the control of cancer progression was suspected but remained inconclusive for many years due to the lack of convincing evidence of a direct link between cancer development and lower immune competence in individuals who succumb to cancer. The polarization of the immune response depends on the microenvironment [93, 94]. Additionally, as mentioned previously tumour cells are able to respond to IL-2, and these cells produce factors that enhance their growth and help them evade or regulate the immune response. In this context, we need to address the following question. Do immune cells attack or help the tumour? Premalignant and early tumour lesions are generally well infiltrated with immune cells (largely T lymphocytes and antigen-presenting cells such as macrophages and DCs, although B cell formations resembling lymphoid follicles are sometimes present) [95, 96]. Of these immune cells, tumour-infiltrating lymphocytes (TILs) are characterized by an inverted and decreased relationship of CD4^+^/CD8^+^ with a reduced capacity to proliferate and an imbalance in the pattern of proinflammatory and regulatory cytokines. These cells produce fewer proinflammatory molecules, such as IL-2 and INF-*γ*, and more regulatory molecules, such as cytokines IL-4, IL-5, IL-10, TGF-*β*, [96–98], and adenosine. Additionally, TILs express inhibitory receptors such as cytotoxic T lymphocyte antigen 4 (CTLA-4) and some of these TILs are anergic [99, 100]. Moreover, tumour cells produce regulatory cytokines, secrete ligands such as MICA and MICB to avoid the activation of cytotoxic cells, and can decrease human leukocyte antigen (HLA) expression to evade immune recognition. Thus, the regulatory molecules produced by TILs and tumour cells induce a regulatory microenvironment that promotes tumour growth and immune system evasion [99, 101]. In this context, we speculate that it is possible that the immune cells help the tumour grow.

Furthermore, important cytokines involved in immune response activation are necessary for tumour elimination (i.e., IL-2 and IFN-*γ*). IL-2 is the fundamental element in the proliferation and activation of cytotoxic cells such as NK and CD8^+^ cells. These cells are able to produce IFN-*γ* and therefore promote the activation and recruitment of other cytotoxic cells. IFN-*γ* has been shown to induce apoptosis in cervical tumour cells and results in increased expression of HLA class I and HLA class II molecules, which significantly enhance the recognition and lysis of tumour cells by specific cytotoxic T lymphocytes (CTLs) activated by CD80-expressing tumour cells [102]. However, other subpopulations of T cells also respond to IL-2 and IFN-*γ*. One of these subpopulations is the *γδ* T cells, which are very important in tumour surveillance because they can kill various types of solid tumours, such as colon cancer, prostate cancer, renal cancer, and cervical cancer [103, 104]. Recently, these cells have become important for cancer immunotherapy because they are considered cytotoxic cells that can lyse tumour cells using perforin and antibody-dependent cellular cytotoxicity. Their importance in cervical cancer immune therapy was noted by Li et al. who demonstrated the presence of *γδ* T cells in cervical cancer samples and found marked cytotoxicity in some cervical cancer cell lines. Additionally, Lertworapreecha et al. found that normal *γδ* T cells treated with pamidronate had the ability to kill cervical cancer cells by inducing apoptosis; this result indicated that many cytotoxic subpopulations were able to respond to IL-2 [103–105].

Altogether, these data pieces support the importance of IL-2 in the antitumour response; however, IL-2 is also necessary for the development of Tregs [33]. It is clear that Treg cells may abrogate NK cell cytotoxicity through the TGF-*β* pathway. Additionally, Tregs are responsible for attenuating antitumour immune responses by suppressing effector T cell proliferation and cytokine production and functionally suppressing the activation and proliferation of CD4^+^ and CD8^+^ effector T cells. Therefore, Treg cell elimination may enhance cytotoxic cell activity and represent a novel therapeutic strategy to prevent the growth or trigger the regression of cervical cancer [95, 106, 107]. The IL-2 concentration is important for the activation and polarization of the immune system. Bich-Thuy et al. showed that high concentrations of IL-2 induced the proliferation of human resting T cells in the absence of other activation signals [108]. Besser et al. observed that TIL cultures from patients with metastatic melanomas growing in the presence of very high IL-2 concentrations (600–6000 IU/mL) expanded massively and secreted IFN-*γ* in response to antigenic stimulation; however, these cells exhibited low direct cytotoxicity. In contrast, TIL cultures grown in the presence of low concentrations of IL-2 throughout the rapid expansion phase expanded to a lower extent and barely secreted IFN-*γ* but displayed high cytotoxic activity [109]. Finally, there are data supporting the hypothesis that IL-2 can prevent and block anergic T cells* in vivo* and* in vitro* [110, 111]. This response might be the same in TILs from patients with cervical cancer, but this conjecture remains to be proven. Therefore, it is necessary to analyse the response of lymphocytes and cervical cancer cells treated with different concentrations of IL-2.

Nevertheless, new therapeutics that affect tumour cells and activate the immune system are needed because recognition of some tumour cells can lead to immune selection. Generally, tumour cells are antigenically indistinguishable from normal cells although genetic abnormalities are already present. Some TILs are able to recognize tumour cells that may be genetically unstable, and some exposed antigens are recognized by the host immune system as foreign and are eliminated. However, this process can lead to the immune selection of tumour cells and promote the permanence and outgrowth of tumour cells that are genetically altered but resistant to immune detection systems. For this reason, the immune system involved in host protection from malignancy plays a dual role and may be dependent on the characteristics of the individual tumour that can help the tumour generate metastases. These cells have unique characteristics, including the ability to penetrate the endothelium and acquire mobility within tissues and lymphatic or blood vessels, thereby making the cells more aggressive; therefore, tumour cells might be more successful in subverting the microenvironment, including immune cells, to subserve the tumour's needs [95]. In this context, IL-2 acts on the tumour cells to inhibit their proliferation and induces activation and IFN-*γ* secretion by immune cells. Therefore, both IL-2 and IFN-*γ* have direct inhibitory effects on tumour cells.

## 7. Concluding Remarks

IL-2 is a pleiotropic cytokine with a multitude of target cells including cancer cells. Therefore, a more detailed inspection of IL-2 signalling in cancer cells will aid in our understanding of the specific effects on this cytokine on cervical cancer cells.

Based on the opposing roles of different concentrations of IL-2 on normal lymphocytes and tumour cells that express the IL-2R, we propose that these differences could be used to develop strategies to treat tumours with these characteristics. For example, vectors could be used to carry high concentrations of IL-2 to the tumour cells without targeting normal lymphocytes to aid in the treatment of cervical cancer.
